# Survival and Toxicities of Metastatic Colorectal Cancer Patients Treated with Regorafenib before TAS-102 or Vice Versa: A Mono-Institutional Real-Practice Study

**DOI:** 10.3390/jcm12020596

**Published:** 2023-01-11

**Authors:** Alessandro Ottaiano, Mariachiara Santorsola, Francesco Perri, Vincenza Granata, Marco Cascella, Francesco Sabbatino, Guglielmo Nasti

**Affiliations:** 1 SSD Innovative Therapies for Abdominal Metastases, Abdominal Oncology, Istituto Nazionale Tumori di Napoli, IRCCS “G. Pascale”, 80131 Naples, Italy; 2 Oncology Unit, Department of Medicine, Surgery and Dentistry, University of Salerno, 84081 Salerno, Italy

**Keywords:** TAS-102, regorafenib, chemotherapy, metastatic colorectal cancer, prognosis

## Abstract

Introduction: Regorafenib and TAS-102 are two orally-administered drugs used to treat refractory metastatic colorectal cancer (mCRC). This study was performed to explore any differences between different therapy sequences: TAS-102 first or regorafenib first. Patients and methods: This is a retrospective and real-practice study in mCRC patients treated according to the ESMO guidelines. They received TAS-102 first (regorafenib second, TR) or regorafenib first (TAS-102 second, RT) at standard doses. Responses to therapy and toxicities were evaluated by RECIST and CTCAE v4.0, respectively. Associations between clinical and pathologic variables and different therapy sequences were evaluated by χ^2^-test. *p* <0.05 was considered statistically significant. A description of any differences in overall survival (OS) between TR and RT was the primary outcome. OS curves were depicted through the Kaplan–Meier product limit. All statistical analyses were performed by the Excel software and MedCalc^®^ version 20.112. Results: Sixty-five patients were analyzed. Twenty-eight received regorafenib before TAS-102, 37 vice versa. Responsiveness to first-line chemotherapy as well as disease control were not different between RT and TR patients. G4 toxicities were very rare. The three most common G1/G2 toxicities with regorafenib were fatigue, anemia, and cutaneous rash; anemia, fatigue, and neutropenia with TAS-102. Compliance to treatment was lower in TAS-102 patients compared to regorafenib. Interestingly, analysis of OS showed a significant difference at Log Rank test (*p* = 0.0366) in favor of TR (median OS: 4.5 months) compared to RT (median OS: 3.0 months; HR: 0.55; 95% CI: 0.31–0.96). Conclusions: we found a significant difference in terms of survival in favor of the TR sequence of treatment. Larger studies are needed to confirm these data and explore specific biomarkers predicting the correct sequence of oral drugs in the treatment of refractory mCRC patients.

## 1. Introduction

Colorectal cancer (CRC) is the third leading cause of cancer-specific death worldwide [1]. The treatment of metastatic CRC (mCRC) has been improved in the last two decades with the introduction of chemotherapy doublets (fluorouracile plus oxaliplatin, fluorouraicle plus irinotecan) and associations of chemotherapy with biologic treatments (anti-angiogenics, anti-EGFR, multi-kinases inhibitors) [2]. However, the prognosis is still unsatisfactory and the survival of mCRC patients rarely surpasses 30 months.

In the last few years, oral drugs (both chemotherapy and biologics) have appeared in the therapeutic scenario of mCRC. Oral administration compared to the intra-venous route exhibits superior patient compliance and non-invasiveness; these characteristics are particularly advantageous in heavily pre-treated patients and later lines of treatment [3]. Regorafenib and TAS-102 are two orally-administered drugs recently introduced in the therapeutic scenario of mCRC.

TAS-102 is a mixture of trifluridine and tipiracil hydrochloride in a molar ratio of 2:1. Tipiracil is a potent inhibitor of thymidine phosphorylase, which rapidly degrades trifluridine; thus, it markedly increases the bioavailability of trifluridine. Trifluridine is a thymidine-based nucleoside derivative. After entry into tumor cells, it is phosphorylated by thymidylate kinase in trifluoromethyl deoxyuridine 5′-triphosphate (F3dTTP). The last is incorporated into DNA, producing DNA strand break and cell death [4]. Most importantly, TAS-102 displays antitumor activity against fluorouracil resistant tumors [5]. Regorafenib is a small molecule able to bind and inhibit multiple signaling pathways including vascular endothelial growth factor receptors (VEGFRs) 2 and 3, KIT, PDGFR, RET and Raf kinases. These pharmacodynamics lead to the inhibition of proliferation and tumor-associated angiogenesis in CRC cells [6]. 

Both TAS-102 and regorafenib are indicated for the treatment of mCRC patients pre-treated with chemotherapy (fluorouracile, oxaliplatin, irinotecan) and biologic therapies (including anti-VEGF (Vascular Endothelial Growth Factor), and anti-EGFR (Epidermal Growth Factor Receptor) in RAS wild-type tumors) based on results from randomized phase III trials compared with placebo [7,8,9]. 

There are neither molecular nor clinical characteristics guiding the use of TAS-102 or regorafenib first in the late treatment of mCRC patients. Lacking head-to-head comparisons, the present study was undertaken to explore any differences between the efficacy of two different therapy sequences including TAS-102 or regorafenib first (TAS-102 before regorafenib versus regorafenib before TAS-102) in a real practice clinical cohort treated in a single, academic, and high-volume oncological center.

## 2. Patients and Methods

### 2.1. Data Sources

This is a retrospective and real-practice study. Data were extracted from a database reporting the clinical information of mCRC patients treated at the Department of Abdominal Oncology (Structure of Innovative Therapies for Abdominal Metastases) of the Istituto Nazionale Tumori di Napoli (National Cancer Institute). Patients were treated according to the ESMO guidelines [10]. Therapies beyond second-line were applied at oncologist discretion since neither internal nor international guidelines were available. The criteria for patients’ inclusion in this cohort were: sequential treatment with both TAS-102 and regorafenib, age <80 years, life expectancy of at least 3 months, adequate renal, liver, and cardiac functions, no death within two months from start of the drug. The latter criterion was adopted to avoid clear and strong prognostic interferences of highly resistant and rapidly progressive disease. Oligo-metastatic disease was excluded. The primary outcome of the study was the description of overall survival (OS), secondary objectives were description of activity, toxicity, and treatment compliance. The study was conducted according to the Declaration of Helsinki and all patients gave signed informed consent before administration of any treatments included in this article. 

### 2.2. Drugs and Schedules

Patients were treated with TAS-102 first (TR) or regorafenib first (RT) from January 2019 to October 2022. The sequence was applied at oncologist discretion lacking well-recognized clinical and/or biological criteria. They received TAS-102 at standard doses of 35 mg/m^2^ twice daily on days 1–5 and 8–12 every 28 days or regorafenib with a dose-escalation approach (starting dose 80 mg/day orally with weekly escalation, per 40 mg increment, to 160 mg/day regorafenib) if no significant toxic events occurred [11]. 

### 2.3. Patients Management

The response to therapy was evaluated by RECIST (Response Evaluation Criteria In Solid Tumors). Total body computed tomography (CT) scan was carried out every 3 months or (if feasible) anticipated in case of clinically evident progression. Complete response (CR) was defined as complete disappearance of all detectable evidence of disease on total body computed tomography. Partial response (PR) was defined as at least a 30% decrease in the sum of diameters of target lesions. Stable disease (SD) was defined as everything between 30% decrease and 20% growth of tumor size. Progressive disease (PD) was defined as at least a 20% increase in the sum of diameters of target lesions. Toxicity was classified according to Common Toxicity Criteria for Adverse Events (CTCAE) v4.0. 

### 2.4. Statistical Analyses and Data Presentation

This is a predominantly descriptive study. Associations between clinical and pathologic variables, toxicities, and different therapy sequences were evaluated by χ^2^-test. *p* < 0.05 was considered statistically significant. Assessment and description of any differences in Overall Survival (OS) between two different treatment sequences (TAS-102 first, regorafenib second vs regorafenib first, TAS-102 second) was the primary outcome. It was measured from the start of the oral drug administration until death from any cause. Progression-free survival was not considered as a study objective because, in some cases, radiologic monitoring after clear evidence of clinical progression was not carried out or in other cases it was conducted outside our Institute. In this context, the vital status is the most solid and reliable outcome to analyze. The Kaplan–Meier product limit method was applied to depict OS curves. Statistical significance at univariate analysis was assessed with a two-tailed Log-Rank test. The risk of death is expressed as HR (Hazard Ratio), which is the risk of death, at any time, for a patient undergoing TR or RT. No attempts were made to perform a multivariate analysis. HR is reported with 95% confidence intervals (CI). All statistical analyses were performed by the Excel software and MedCalc^®^ version 20.112 (edCalc Software Ltd, Ostend, Belgium).

## 3. Results

### 3.1. Patients’ and Treatments Characteristics

Sixty-five patients affected by mCRC received late lines of treatment with regorafenib and TAS-102 from 2019 to 2022. Twenty-eight received regorafenib before TAS-102 (RT), 37 vice versa (TR). Patients’ and treatments characteristics are shown in Table 1. Ages, gender, histology, and side were homogeneously distributed between patients’ subsets. ECOG Performance Status at the start of the sequence was better in TR compared to RT (42.8% in RT group had PS 2 *vs* 16.2% in TR, *p* = 0.036). Although not significant, patients treated with TAS-102 first (compared to regorafenib first) were more likely to have a higher number of metastatic sites (>2 sites: 62.2% *vs* 39.2% in regorafenib first), previous treatment lines (>3: 67.6% *vs* 42.8% in regorafenib first) and RAS mutated tumors (K- or N-RAS mutations: 64.9% *vs* 42.8% in regorafenib first). Responsiveness to first-line chemotherapy as well as DC (diseases control) were not different between RT and TR patients.

### 3.2. Safety and Compliance

Adverse events of any grades per patient according to different sequence therapies with their relative frequencies are reported in Table 2. G4 toxicities were rare accounting for a high manageability of these drugs in late lines of treatment. The three most common G1/G2 toxicities with regorafenib were fatigue, anemia, and cutaneous rash. The three most common G1/G2 toxicities with TAS-102 were anemia, fatigue, and neutropenia. There were not statistically significant differences in the type of toxicity for the same drug according to different therapy sequences. Compliance to treatment is described in Table 3. It was lower in TAS-102 patients compared to regorafenib. In fact, 55.4% and 78.5% (23.1%) of patients who received regorafenib and TAS-102, respectively, underwent dose reductions. Treatment delays higher than 2 weeks were more frequent in TAS-102-treated patients (29.2% *vs* 18.5% in regorafenib).

### 3.3. Survival Analysis

**Figure 1:**
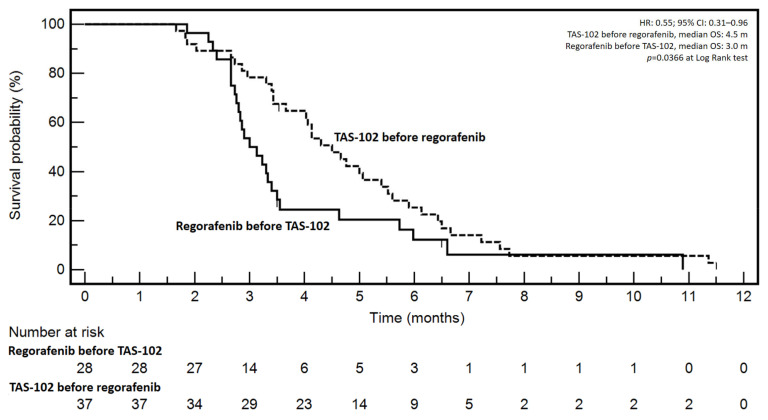
Kaplan–Meyer survival curves according to different sequences therapy.

**Figure 2:**
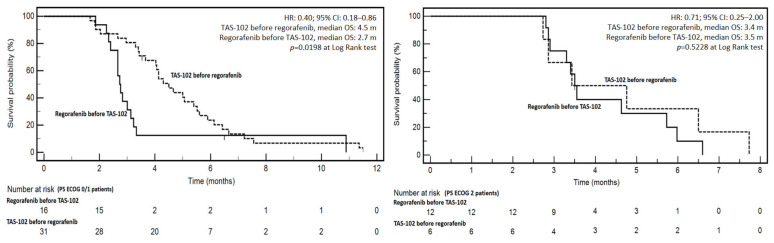
Kaplan–Meier survival curves according to TAS-102 first or regorafenib first in PS ECOG 0/1 (left side) or PS ECOG 2 patients (right side).

Considering the retrospective nature of the study and the unbalanced patients’ characteristics between treatments (see Patients’ and treatments characteristics section), a descriptive analysis of patients’ overall survival (OS) in RT and TR sequences was carried out without attempts to perform multivariate analysis. Interestingly, a significant difference at Log Rank test (*p* = 0.0366) emerged in favor of TR (TAS-102 before regorafenib, median OS: 4.5 months *vs* 3.0 months in RT; HR: 0.55; 95% CI: 0.31–0.96) (Figure 1). Since there was significant unbalancing in PS ECOG of patients treated with different sequences, survival was differentially analyzed in PS ECOG 0/1 and 2 patients (Figure 2). Interestingly, the positive effect of TR vs RT was confirmed in the PS ECOG 0/1 patients (HR: 0.40; 95% CI: 0.18–0.86; median OSs TR *vs* RT: 4.5 *vs* 2.7 months; *p* = 0.0198) but lost in the PS ECOG 2 ones (HR: 0.71; 95% CI: 0.25–2.00; median OSs TR *vs* RT: 3.4 *vs* 3.5 months; *p* = 0.5228).

## 4. Discussion

We performed an analysis of a “real-practice” clinical cohort treated in a single, academic and referral oncologic center to inform the scientific community about any differences in toxicities and/or efficacy of two different therapy sequences based on the use of two well-known oral drugs (TAS-102 and regorafenib) in the late treatment of mCRC patients. No prospective, randomized studies have been so far reported about this issue. In fact, it is reasonably impractical to perform large randomized controlled trials in such a clinical setting. 

A previous retrospective analysis of time-to-outcome (progression-free and overall survival) in a real-practice mCRC cohort treated with at least two prior regimens, showed no statistically significant differences between the two sequences (TAS-102/regorafenib, 29 patients, vs. regorafenib/TAS-102, 24 patients), although patients switching to TAS-102 had a better outcome [12]. However, differently from our series, very few patients had PS ECOG 2 at the start of late therapies (4 patients) that makes the “real-practice” nature of this clinical series questionable. By contrast, in another study, the strategy of treatment with regorafenib before TAS-102 (16 patients) versus vice versa (6 patients) was associated with better survival. However, unbalances in PS and disease burden favoring regorafenib first could be responsible of these results as discussed by the authors [13]. Furthermore, in a large retrospective cohort study collecting data from 269 diagnosis procedure combination (DPC) hospitals in Japan, 7279 mCRC patients were identified and analyzed. Interestingly, an analysis of regorafenib first (2474 patients) versus TAS-102 first (4805 patients) subgroups showed a slight but significant survival advantage for TAS-102 first (11.9 *vs* 11.1 months; HR: 0.90; 95% CI: 0.83–0.96; *p* = 0.002). Propensity score-matching and sensitivity analyses confirmed the superiority of TAS-102. However, some limitations affected results interpretation and translation into clinical practice including patients’ ethnicity, absence of information on sequences, PS, RAS/BRAF mutational status, and response assessments [14].

In our clinical cohort, we found a significant difference in terms of survival for TAS-102 when it is used before regorafenib. The unbalance of PS ECOG 0/1 patients in favor of TR (TAS-102 first) does not account for this effect since these results were confirmed when excluding PS ECOG 2 patients. Furthermore, it is noteworthy that patients treated with TAS-102 in our cohort had a higher burden of disease (Table 1). Interestingly, in vitro and in vivo experiments in CRC cell lines treated with different sequences of TAS-102 and regorafenib showed greater anti-tumor effects when TAS-102 was followed by regorafenib in CRC cells [15]. However, still to date, there are neither clinical nor molecular biomarkers guiding the choice of TAS-102 before regorafenib, or vice versa, in the treatment of late phases of mCRC patients. 

Our study has some limitations including its retrospective nature, the relatively small size and the absence of randomization. Thus, unknown patients’ selection biases could affect results that must be interpreted as hypotheses-generating. However, it deserves to be emphasized that the clinical characteristics of our cohort paradoxically reinforce the eventual positive effect of TAS-102 first versus regorafenib first. 

## 5. Conclusions

We provide evidence that TAS-102 before regorafenib may be associated with better survival in refractory mCRC patients. Future studies in larger series including the exploration of specific biomarkers are needed to definitively address any differences in the efficacy, as well as in the biological and genetic trajectories eventually associated with these different therapeutic sequences. 

## Figures and Tables

**Table 1:** Clinico-pathological characteristics of patients.

Variable	No.	%	RT (No.)	TR (No.)	*p*
Age					
Median (year)	63		63	65	
Range (year)	41–73		41–70	43–73	
<65 years (No.)	29	44.6	13	16	
≥65 years (No.)	36	55.4	15	21	0.7996
Gender					
Male	32	49.2	13	19	
Female	33	50.8	15	18	0.6965
Histology					
Classical adenocarcinoma	59	90.8	26	33	
Mucinous adenocarcinoma	6	9.2	2	4	0.6157
Side					
Right	42	64.6	19	23	
Left	23	35.4	9	14	0.6371
PS ECOG					
0	2	3.1	0	2	
1	45	69.2	16	29	
2	18	27.7	12	6	0.0362
No. of metastatic sites					
1	8	12.3	3	5	
2	23	35.4	14	9	
>2	34	52.3	11	23	0.0970
K- or N-RAS					
Wild-type	29	44.6	16	13	
Mutated	36	55.4	12	24	0.0795
BRAF					
Wild-type	60	92.3	26	34	
Mutated	5	7.7	2	3	0.8859
No. of previous treatment lines					
2	28	43.1	16	12	
3	27	41.5	7	20	
4	10	15.4	5	5	0.0580
Response to first-line chemotherapy					
DC	59	90.8	26	33	
Not DC	6	9.2	2	4	0.6157
Response to regorafenib *					
DC	2	16.7	2	0	
Not DC	10	83.3	9	1	0.6547
Response to TAS-102 **					
DC	10	37.1	1	9	
Not DC	17	62.9	4	13	0.3911

* Sixteen patients treated with regorafenib (in RT and TR) did not undergo to radiologic reassessment of disease. ** Ten patients treated with TAS-102 (in RT and TR) did not undergo to radiologic reassessment of disease.

**Table 2:** Hematologic and non-hematologic toxic events per patient.

	Regorafenib			TAS-102		
Toxicity	R→T (No,%/28)	T→R (No,%/37)	*p*	T→R (No,%/37)	R→T (No,%/28)	*p*
Alopecia						
G1/G2	7 (25.0)	5 (13.5)		12 (32.4)	15 (53.6)	
G3	2 (7.1)	2 (5.4)	0.77	4 (10.8)	4 (14.3)	0.78
Anemia						
G1/G2	19 (67.8)	22 (59.5)		25 (67.6)	26 (92.8)	
G3	2 (7.1)	3 (8.1)	0.79	6 (16.2)	6 (21.4)	0.95
AST/ALT increase						
G1/G2	9 (32.1)	8 (21.6)		12 (32.4)	15 (53.6)	
G3	2 (7.1)	0 (0.0)	0.21	1 (2.9)	2 (7.1)	0.71
Fatigue						
G1/G2	23 (82.1)	24 (64.9)		24 (64.9)	23 (82.1)	
G3	4 (14.3)	6 (16.2)	0.61	6 (16.2)	5 (17.9)	0.83
Diarrhea						
G1/G2	7 (25.0)	9 (24.3)		11 (29.7)	13 (46.4)	
G3	1 (3.6)	2 (5.4)	0.74	3 (8.1)	4 (14.3)	0.89
Hypertension						
G1/G2	11 (39.3)	12 (32.4)		2 (5.4)	3 (10.7)	
G3	1 (3.6)	0 (0.0)	0.31	0 (0.0)	0 (0.0)	NA
Nausea/Vomiting						
G1/G2	4 (14.3)	6 (16.2)		13 (35.1)	12 (42.8)	
G3	0 (0.0)	1 (2.7)	0.44	1 (2.7)	2 (7.1)	0.54
Neutropenia						
G1/G2	4 (14.3)	6 (16.2)		18 (48.6)	13 (46.4)	
G3	1 (3.6)	2 (5.4)		7 (18.9)	9 (32.1)	
G4	0 (0.0)	0 (0.0)	0.84	1 (2.7 per patient)	0 (0.0)	0.97
Rash						
G1/G2	16 (57.1)	14 (37.8)		1 (2.7)	2 (7.1)	
G3	4 (14.3)	6 16.2)	0.47	0 (0.0)	0 (0.0)	NA
Stomatitis						
G1/G2	12 (42.8)	15 (40.5)		14 (37.8)	12 (42.8)	
G3	3 (10.7)	2 (5.4)	0.52	2 (5.4)	2 (7.1)	0.88
Thrombocytopenia						
G1/G2	2 (7.1)	1 (2.7)		12 (32.4)	15 (53.6)	
G3	0 (0.0)	1 (2.7)	NA	3 (8.1)	4 (14.3)	0.94

**Table 3:** Compliance to treatments.

TAS-102		
Dose Reductions	No.	%
Ab initio (−5 mg/m^2^/dose)	16	24.6
During treatment	35	53.8
−5 mg/m^2^/dose	2	
−10 mg/m^2^/dose	27	
−15 mg/m^2^/dose	6	
No dose reduction (100% dose intensity)	14	21.5
Treatment delays (>2 weeks)	19	29.2
**Regorafenib**		
Ab initio * (80 mg/die)	20	30.8
During treatment (−40 mg/dose)	16	24.6
No dose reductions (100% dose intensity)	29	44.6
Treatment delays (>2 weeks)	12	18.5

* The dose has not been escalated.

## Data Availability

Most data are under privacy restrictions. A representative dataset is reported in https://zenodo.org/record/7466934#.Y7kp3NWZND8.
